# Castleman disease and paraneoplastic pemphigus in a pregnant woman: A case report

**DOI:** 10.1097/MD.0000000000024990

**Published:** 2021-04-02

**Authors:** Beibei Cui, Hui Lin

**Affiliations:** Department of Rheumatology and Immunology, West China hospital, Sichuan University, Chengdu, Sichuan Province, PR China.

**Keywords:** Castleman's disease, mucosal ulcers, paraneoplastic pemphigus

## Abstract

**Rationale:**

Orogenital ulcers can be observed in various conditions, such as Behcet disease, infection and also paraneoplastic pemphigus (PNP). Castleman disease (CD), which is a rare cause of paraneoplastic pemphigus represents a heterogenous lymphoproliferative disorder of unknown etiology. Paraneoplastic pemphigus associated with CD in pregnancy is rare and has not been reported yet.

**Patient concerns:**

We report a rare case of CD in a 26-year-old pregnant woman with orogenital ulcers. The patient suffered from mucosal erosions and uveitis at 23 weeks of gestation. A retroperitoneal mass (9.7×7.3×11.8 cm) was identified by CT scan.

**Diagnoses:**

According to histological and immunohistological findings, a diagnosis of unicentric CD, hyaline vascular type, and PNP was formulated.

**Intervention:**

High dose methylpredisonlone was given for the therapy. Pancreatic uncinatectomy, portal vein and superior mesenteric vein repair, pancreaticojejunostomy, and caesarean section were performed on the patient to remove the tumor and the fetus.

**Outcomes:**

The fetus did not survive after surgery. The patient did not achieve remission and she died from epidermolysis and sepsis several months later.

**Lessons:**

PNP associated with CD is a rare lymphoproliferative disorder and needs to be differentiated from other orogenital diseases by histological features.

**Ethics and dissemination:**

Written informed consent was obtained from the patient for publication of this case report and accompanying images. Ethical approval of this study was granted by the Ethics Committee of West China Hospital of Sichuan University. (Ethics Reference No: 2021143).

## 1 Introduction

Paraneoplastic pemphigus (PNP) is a rare autoimmune, bullous disease, characterized by painful mucosal ulcerations and polymorphous skin lesions.^[1]^ Castleman disease (CD) represents a heterogenous lymphoproliferative disorder of unknown etiology.^[2]^ The association of PNP and CD is very rare. Here we report a pregnant woman who was diagnosed with PNP and CD based on histological and immunohistological findings. The patient has provided informed consent for publication of the case.

## 2 Case report

**Figure 1:**
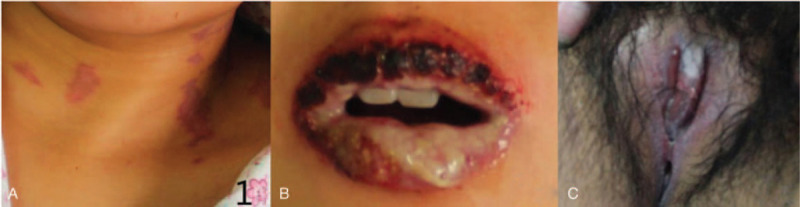
Erythema on patient's neck (A), multiple ulcers in oral cavity (B) and genital ulcers (C).

A 26-year-old woman in her 23-week of gestation, was admitted to our rheumatology and immunology department with a 4-week history of oral and genital ulcers as well as bilateral uveitis in September 2015 (Fig. 1A–C). Due to multiple painful ulcers, the patient could not open her mouth widely. Erythema developed on her neck and breasts. Nikolsky sign was negative and bullae were not found. She has no significant past medical history and had delivered a healthy baby 5 years before admission. Abnormal laboratory findings included positive antinuclear antibody (1:320), elevated immunoglobulin (IgA 3.6 g/L, IgG17.1 g/L, IgE 208.9IU/mL) and elevated IL-6 (43.46 pg/mL). Lactate dehydrogenase (LDH) levels were within normal range (144 IU/L). DNA of cytomegalovirus was 3.30E + 02 copies/mL. Tests for HIV, HBV, syphilis, and HCV infection were negative. Sonography showed single live fetus in uterus (HCG>1000 mIU/mL). Due to possible radiation injury, the patent declined further image examinations. For lack of image examination, The patient was diagnosed with Behcet disease and methylprednisolone (80 mg per day) was given. 7 days later, because of non-remission, the dose of methylprednisolone was changed into 200 mg per day for 3 days, however, the symptoms got even worse.

**Figure 2:**
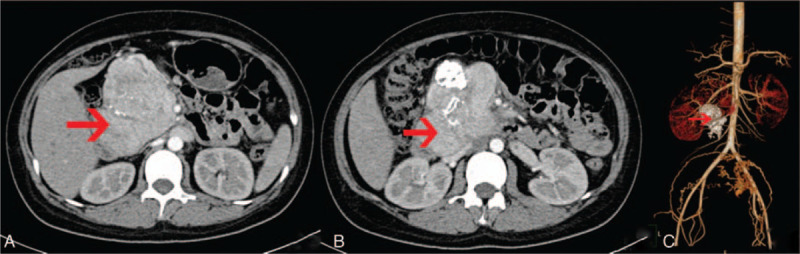
CT scan of the abdomen showing a highly vascularized retroperitoneal tumor measuring 9.3 × 7.3 × 11.8 cm with intratumor calcifications (A, B) and three-dimensional reconstruction CT of abdominal vessels and the tumor (C). No other tumor localization in the abdomen nor enlarged lymph nodes were detected.

**Figure 3:**
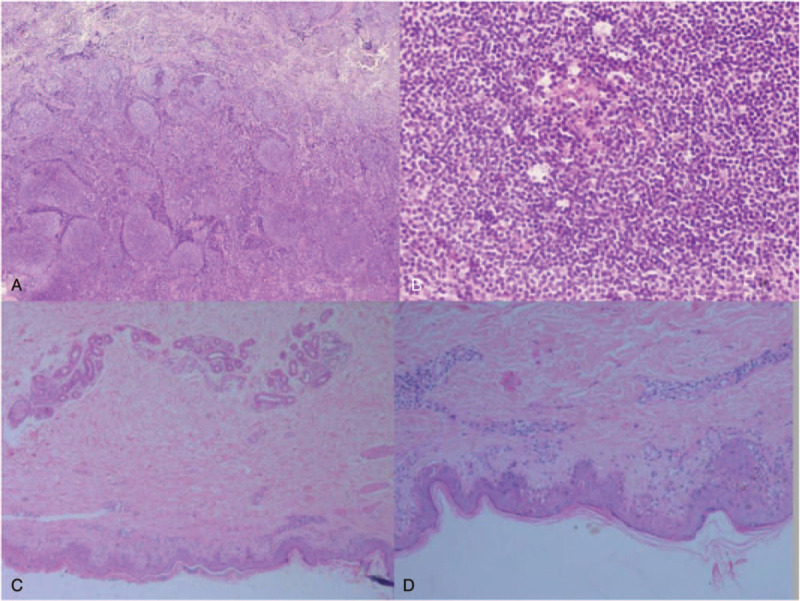
Hematoxylin-eosin-stained histopathological images. Histological examination of the mass revealed a Castleman tumor of hyaline vascular type (A-B): Low-power view showed increased numbers of lymphoid follicles with germinal centers and small vessels. The interfollicular areas demonstrate obliteration of medullary sinuses. (A, H&E, ×40); Higher magnification revealed proliferation of vascular endothelial cells and concentric arrangement of mantle zone lymphocytes in an “onion skin” pattern. Interfollicular areas demonstrate angiogenesis, scattering with plasma cells (B, H&E, ×200). Histopathological examination of cutaneous lesional specimen (C-D): extensive liquefaction of basal cells and perivascular lymphocytic infiltration in upper dermis. (C, H&E, ×40; D, H&E, ×100).

Further imaging were arranged. A CT scan of the abdomen showed a tumor (9.7 × 7.3 × 11.8 cm) with intratumor calcification at the root of mesentery below the head of pancreas (Fig. 2AC). In 29 weeks of gestation, pancreatic uncinatectomy, portal vein and superior mesenteric vein repair, pancreaticojejunostomy, and cesarean section were performed. The fetus didn’t survive after surgeries. Pathological examination of the specimen revealed an enlarged lymph node showing changes indicative of CD, predominately the hyaline vascular type (Fig. 3A–B). Histopathological examination of cutaneous lesional specimen revealed extensive liquefaction of basal cells and perivascular lymphocytic infiltration in upper dermis (Fig. 3CD). Direct immunofluorescence (DIF) evaluation showed intercellular deposition of IgG (+) among keratinocytes and linear deposition of IgG(+), C3(+), IgM(−), IgA(−) along basement membrane. The histopathological findings of cutaneous specimen were consistent with PNP.

After excision of the tumor, LDH levels (211IU/L) and C reactive protein levels (139 mg/L) increased, while IL-6 (21.54pg/mL) decreased. The patient declined further examinations and chemotherapy. Unfortunately, mucocutaneous lesions continued to spread more widely. Three month later, the patient died from epidermolysis and sepsis.

## 3 Discussion

Our patient represents a rare case of localized hyaline vascular type CD associated with PNP. It is interesting to speculate about the characteristics of the two associated diseases.

CD is a rare lymphoproliferative syndrome which was first described in 1954.^[1]^ Based on histopathology and pathogenesis, CD can be divided into hyaline vascular (HV) type, plasma cell (PC) type and mixed type. ^[2]^ A few cases of CD during pregnancy has been reported. As our case, all of these cases demonstrated localized hyaline-vascular type CD. Hyperplastic lymphoid follicles and atrophic germinal center are main characteristics. In the pathogenesis of CD, IL-6 has been considered as a crucial trigger for CD.^[3]^ In the pathogenesis of CD, IL-6 has been considered as a crucial trigger for CD. ^[4–6]^ Dysregulated and overproduced IL-6 stimulates the production of acute phase reactants, resulting in constitutional symptoms of CD. IL-6 also stimulates B-cell proliferation and induces the expression of vascular endothelial growth factor resulting in increased angiogenesis.^[2]^ In our case, IL-6 was 10 times the upper limit of the reference range at the beginning and decreased after tumor excision, which implies it might be related to CD. Besides IL-6, positive antinuclear antibody and elevated immunoglobulin has also been observed in our case. Unlike immunoglobulin, positive antinuclear antibody has only been reported in two CD cases.^[7,8]^ We speculated that abnormal immunoglobulin and antibodies in this case may be the result of overactive immune responses associated CD.

CD accompanied by PNP has only been reported in a few cases.^[9,10]^ PNP is an autoimmune blistering disease linked to a lymphoproliferative disorder as well. The cause of death in patients with PNP is attributed to multiple factors, such as bronchiolitis obliterans, respiratory failure, sepsis, and gastrointestinal bleeding. Skin and mucosal lesions which expand gradually are various, including erythema, blisters, oral and anogenital ulcers, and sometimes Stevens-Johnson-like changes. The role of CD in pathogenesis of PNP remains unclear. The cross-reaction induced by similar antigens, proteins and epitope spreading might explain the autoimmune changes on skin and mucosa.^[11]^ J Wang studied 7 cases of Castleman tumors and found out that clones of B lymphocytes in the CD may have the structural basis for producing autoantibody, so he postulated that Castleman tumor produce antibodies directly.^[12]^

For patients with only orogenital and ocular symptoms, differential diagnosis of PNP could be difficult. Orogenital ulcers, presents in various diseases, such as Behcet's disease, complex aphthous dermatitis, herpes simplex virus infections, inflammatory bowel disease, and Reiter's syndrome.^[13]^ It is difficult to make differential diagnosis by clinical symptoms. Histologically, certain features are reported to be highly specific for PNP, including combined intercellular substance with basement membrane staining on direct immunofluorescence, suprabasal acantholysis keratinocyte necrosis and a lichenoid infiltrated.^[14]^ On the other hand, leukocytoclastic vasculitis and fibrinoid necrosis of postcapillary venules are common pathological features in BD, and lymphocytic perivasculitis were also described in several cases.^[15]^

CD associated with PNP usually predicts poor prognosis. Even after complete resection of neoplasm and intensive therapeutic intervention, progression to death occurs sometimes. Progressive bronchiotisis obliterans and respiratory failure may happen over a period of 2 years.^[10]^ Even so, removing the neoplasm remains the first-line treatment. In the study by Wang, most of the patients’ lesions improved and titer of the circulation auto-antibody decreased after resection of the tumor. similar results were observed in other studies.^[12]^ Besides surgery, chemotherapy and immunomodulation therapy might be available options for autoimmune disorders. Japanese pemphigus treatment guidelines recommend systemic steroid therapy for PNP treatment.^[16]^ Other medications such as azathioprine, cyclosporine, cyclophosphamide, mizoribine, mycophenolated mofetil and rituximab could be added. However, the efficacy of these treatments is equivocal. ^[17]^ Our patient showed poor response to high-dose corticosteroid pulse therapy. In some case reports, cyclosporine did not improve the patients’ mucosal lesions or respiratory condition.^[18]^ In another case, a patient with PNP and B cell lymphoma was given steroid pulse therapy combined with R-CHOP, and rituximab, IVIG, cyclosporine in further treatments. The patient died of infection approximately 6 months after hospitalization.^[19]^ Moreover, radiotherapy and stereotactic radiosurgery, which has been performed successfully in several patients could be future options for treating the combination of these two diseases.^[20]^

## 4 Conclusions

In conclusion, PNP associated with CD is a rare lymphoproliferative disorder, which is characterized by mucocutaneous lesions. Due to lack of abdominal images and results of skin biopsy, the patient was misdiagnosed with Behcet's disease on admission. Although the limited number of cases reported and the lack of prospective studies make it difficult to draw a full picture of differentiations, our case along with the literature review suggests that histological features might be helpful for diagnosing. Resection of neoplasm is a prior choice for patients with PNP associated CD, while chemotherapy and radiotherapy might be used to control autoimmune responses.

## Author contributions

**Conceptualization and project administration**: Hui Lin

**Data curation:** Beibei Cui.

**Writing – original draft:** Beibei Cui.

**Writing – review & editing:** HUI LIN.
